# Partition Enrichment of Nucleotide Sequences (PINS) - A Generally Applicable, Sequence Based Method for Enrichment of Complex DNA Samples

**DOI:** 10.1371/journal.pone.0106817

**Published:** 2014-09-09

**Authors:** Thomas Kvist, Line Sondt-Marcussen, Marie Just Mikkelsen

**Affiliations:** Samplix ApS, Ballerup, Denmark; University of Helsinki, Finland

## Abstract

The dwindling cost of DNA sequencing is driving transformative changes in various biological disciplines including medicine, thus resulting in an increased need for routine sequencing. Preparation of samples suitable for sequencing is the starting point of any practical application, but enrichment of the target sequence over background DNA is often laborious and of limited sensitivity thereby limiting the usefulness of sequencing. The present paper describes a new method, Probability directed Isolation of Nucleic acid Sequences (PINS), for enrichment of DNA, enabling the sequencing of a large DNA region surrounding a small known sequence. A 275,000 fold enrichment of a target DNA sample containing integrated human papilloma virus is demonstrated. Specifically, a sample containing 0.0028 copies of target sequence per ng of total DNA was enriched to 786 copies per ng. The starting concentration of 0.0028 target copies per ng corresponds to one copy of target in a background of 100,000 complete human genomes. The enriched sample was subsequently amplified using rapid genome walking and the resulting DNA sequence revealed not only the sequence of a the truncated virus, but also 1026 base pairs 5′ and 50 base pairs 3′ to the integration site in chromosome 8. The demonstrated enrichment method is extremely sensitive and selective and requires only minimal knowledge of the sequence to be enriched and will therefore enable sequencing where the target concentration relative to background is too low to allow the use of other sample preparation methods or where significant parts of the target sequence is unknown.

## Introduction

Molecular biology methods are increasingly used for diagnosis, prognostication and prediction of disease and efficiency of therapy. While PCR is now widely used, sequence analysis on relevant samples such as swabs, blood or feces needs preparation as the collection of sequence information is complicated by the high level of background DNA from e.g. blood cells or microbial cells [1]. As the target cell or molecule may only be present in a few copies in the complex sample the selectivity of a sample preparation method must be exceptionally high. Next generation sequencing offers extremely high throughput and low cost per sequenced base pair. Samples are typically prepared by generating PCR fragments of a few hundred base pairs, containing adapter sequences at both ends. The fragments are then clonally amplified before the actual sequencing. The small sequence fragments are aligned and a full sequence is constructed. In next generation sequencing, the error rate is typically at least 0.1%, even after stringent filtering based on quality scores and a given mutation must therefore be present in at least 1% of the sequences of a given region for the investigator to be fairly sure that the purported mutation is not a falsely interpreted sequencing error [2]. When the target sequence constitutes less than 1%, enrichment needs to be included in the sample preparation. This can be done using a procedure such as ICE-COLD PCR, but will only retrieve a small fragment containing the mutation [3]. Enrichment of specified regions of a genome can be performed using hybrid capture techniques, typically using biotin-linked capture probes [4], however the selectivity of these techniques is limited and the preparation of capture probe libraries is laborious and expensive.

**Figure 1:**
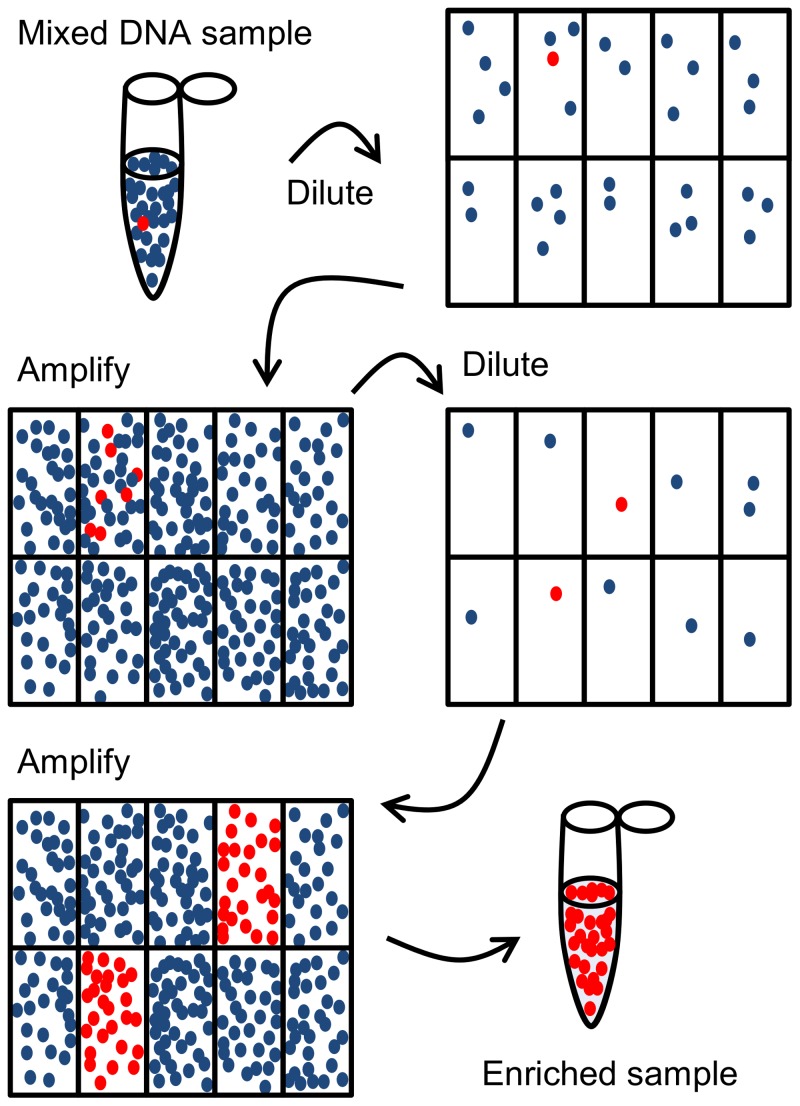
The principle behind the PINS enrichment is a repetition of the steps of end-point dilution and general amplification. The mixed DNA sample is diluted until less than one out of 5–10 wells contain the target DNA molecule to be enriched and the DNA in all wells is then generally amplified using Phi29 polymerase. A sample containing target DNA is selected for next round of dilution and general amplification based on the result of qPCR directed towards the target.

PINS is a deceptively simple and powerful technology for DNA enrichment, only relying on PCR detection of a short specific sequence. It combines terminal dilution, target detection and whole genome amplification (Figure 1). The prerequisite for using the technology is that the target molecule contains a known sequence and that this sequence can be detected in the original sample. The first step of enrichment consists of repeated dilutions of the sample to the point, where the target is no longer present in all wells when the final dilution is partitioned into a number of replicate samples. The distribution of target DNA molecules among these partitions follows Poisson statistics, and at the limiting dilution, most reactions contain either one or zero target DNA molecules [5]. All replicate diluted samples are now amplified, using multiple displacement amplification (MDA), and the presence/absence of the target is detected in the individually amplified wells. The MDA reaction will produce fragments of more than 10,000 base pairs on average [6]. Wells containing a target DNA (positive wells) will have a higher concentration of target fragments relative to background, a principle that is also known from e.g. digital droplet PCR [7], [8]. The ratio of positive to negative wells will determine the degree of target enrichment. This procedure of end point dilution and amplification is now repeated until the desired abundance of positive fragment is achieved, and the sample is ready for downstream applications such as sequencing.

The sensitivity of PINS enrichment is determined only by the sensitivity of detection and is therefore high compared to other means of enrichment such as hybridization-based techniques. In the results section below, it is shown that enrichment can be performed from a starting point of 0.001% integrated human papilloma virus (HPV) copies relative to human background genomes corresponding to less than three target molecules per µg of total DNA. HPV is the principal cause of virtually all cervical cancers [9] and subsets of head and neck cancers [10]. HPV18, which is used in the current study, is the second most prevalent carcinogenic type of HPV, causing around 16% of all cervical cancer cases [11]. PINS enrichment of HPV containing sequences from e.g. a blood sample provides not only the sequence of the virus, but also sequence of the integration site and nearby region.

The PINS enrichment method successfully demonstrated in this study enables sequencing of samples with far lower target to background ratio than previously seen and can be applied to viruses as well as a broad range of other applications through changing only one set of detection primers.

## Materials and Methods

The data were analyzed anonymously and consent was therefore not needed. The leukocytes used for extraction of negative background DNA were kindly donated by Quantibact A/S, Denmark. They were originally provided by Department of Clinical Immunology - Blood Bank (Rigshospitalet, Denmark) where they were discarded for clinical use. The leukocyte sample was part of a sample used in a previous study [12], the sample was fully anonymized and it could not be traced back to the original donor.

### Nucleic acid templates

HeLa DNA containing HPV18 was purchased from New England Biolabs in a concentration of 100 ng/µL (NEB-N4006S). Background DNA was extracted from leukocytes from an HPV18 negative blood sample and DNA was extracted using a DNA Extraction kit (Fermentas – GeneJET^TM^Genomic DNA Purification Kit).

Both templates were pre-amplified using Phi29 amplification (described below) and visually inspected by gel electrophoresis prior to quantification using a Quantous Fluorometer (Promega BioSystems) according to the instructions provided by the manufacturer (Instructions for use of product E6150).

### Phi29 amplification

Phi29 MDA reactions were carried out using the Sampliphi kit as described by the manufacturer (Samplix ApS, Denmark): Two solutions, Mix 1 and Mix 2 were prepared separately.

Mix1: 1 µL DNA template, 1 µL “Solution A”, 2.5 µL freshly prepared “Solution B”, and 0.5 µL nuclease free (NF) water (Sigma-Aldrich) was thoroughly mixed using a vortexer. Then, the mixture was heated to 94°C for 3 minutes and immediately transferred to ice.

Mix2: 4 µL “Solution C”, 2 µL “Solution D”, 8.5 µL NF water, and 0.5 µL (10 U/µL) Phi29 polymerase was added to the reaction.

After combining Mix 1 and 2, the reaction was mixed thoroughly and incubated at 30°C for 16 hours. Finally, the reaction was terminated by heat inactivation at 65°C for 10 minutes. No reactions were observed in any of the included negative controls using NF water (Sigma) as “non-template”.

### Setting up the initial template

A Phi29 amplified sample of HeLa DNA (228 ng/µL) was diluted into Phi29 amplified HPV18-negative background DNA (245 ng/µL) to create an initial sample containing 0.6 target copies per µL in a total DNA concentration of 211 ng/µL.

### Primer design

HPV18 PCR primers were designed using sequence information (GenBank accession number: GQ180790) from GenBank in the primer design application of CLC Main workbench v6 (CLCbio, a Qiagen company) targeting a desired product size of 100–130 bp. Numbers in HPV primer IDs corresponds to the location in nucleotide sequence (GenBank accession number: GQ180790). A total overview of all primers used in this study is listed in Table 1.

**Table 1:** PCR Primers and oligos used for target detection, sequencing and genome walking.

Primer ID	Sequence (5′ – 3′)	Target/Description
HPV5901f	GTTTAGTGTGGGCCTGTGC	Target: HPV18
HPV5994r	GGCATGGGAACTTTCAGTGT	Target: HPV18
HPV6640r	GAGACTGTGTAGAAGCACATATTG	Target: HPV18
HPV6552f	CATAAGGCACAGGGTCATAAC	Target: HPV18
HPV7459f	CCGATTTCGGTTGCCTTTG	Target: HPV18
HPV7819r	CCCAACCTATTTCGGTTGC	Target: HPV18
WalkID	GCGCTGCAGGCATGCGAGCTC	Primer for oligo cassette [14]
WalkOL	GCGCTGCAGGCATGCGAGCTCCCAAGCTTGATCG	Part of oligo cassette
RGW-*Hind*III	AGCTCGATCAAGCTTGGGAGCTCGCATGCCTGCAGCGC	Part of oligo cassette
RGW-*Bgl*II	GATCCGATCAAGCTTGGGAGCTCGCATGCCTGCAGCGC	Part of oligo cassette

### Quantitative PCR (qPCR) conditions

qPCR reactions were performed using BioRad Sso ADV SYBR Green Supermix in the following mixture: 10 µl SSO, 7.4 µL NF water, 0.8 µL (5 µM) HPV5901f, 0.8 µL (6 µM) HPV-5994r, using PCR conditions of: (94°C/65.8°C/72°C) at time intervals of (15sec/15sec/15sec) for 50 cycles.

### Most Probable Number (MPN) Calculations

Determination of the HPV18 target copy number in the samples was based on MPN quantification as described in [13], and all quantifications were carried out as five-tube assays (MPN5). Serial dilutions were carried out as either 5- or 10-fold dilutions and calculated according to the relevant MPN tables. Due to the high content of DNA in the first two series of analysis, evaluation of the PCR product formation was established by agarose gel electrophoresis. Correct size target amplification was 113 bp, and was easily recognizable in a 2% agarose gel. After completion of the two first rounds of amplification, 10 fold dilutions were performed prior to qPCR, thereby minimizing the background fluorescence from the reaction template and agarose gel analysis was only performed as occasional controls in enrichment round 3–6.

### Abundance

The abundance of target relative to total human genomes in the sample was calculated based on the concentration of DNA. Assuming an average molecular weight per base pair of 660 g*mol^−1^*bp^−1^, a human genome size of 3.2 billion base pairs, and using Avogadros constant of 6.022+10^23^ an average of 285 human genomes per ng human DNA is reached. Consequently, the target abundance for the initial sample is:
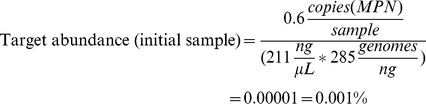
where 0.6 and 211 is the target number (copies/µL) and DNA concentration (ng/µL) respectively (Table 2).

**Table 2:** Quantification of results from rounds 0–6 of the PINS enrichment of HPV18 containing DNA.

Round of enrichment	DNA conc.	Target number^1^	Target number^2^	Increase^3^	Total Increase^4^	Increase^3^	Total increase^4^	Abundance^5^
	ng/ µL	per µL	per ng	per µL	per µL	per ng	per ng	%
0	211	0.6	0.0028	n.a.	n.a.	n.a.	n.a.	0.001
1	234	30	0.1	50	50	45	45	0.04
2	219	170	0.8	5.7	283	6.1	273	0.27
3	165	540	3.3	3.2	900	4.2	1,151	1.15
4	186	2,800	15.1	5.2	4,667	4.6	5,294	5.28
5	112	14,000	125	5.0	23,333	8.3	43,958	43.8
6	42	33,000	786	2.4	55,000	6.3	276,310	275

1 Number of targets per µL of total DNA in the sample as determined by MPN,

2 Number of targets per ng of total DNA in the sample as determined by MPN,

3 Increase in target number relative to the previous round of enrichment,

4 Increase in target number relative to initial sample (round 0),

5 Number of targets per human genome (3.2 billion base pairs).

### Rapid Genome Walking (RGW)

Sequencing of the PINS enriched sample was carried out as described by Kilstrup and Kristiansen [14]. In brief, 3 µL Phi amplified sample (fifth enrichment) was digested by fast digest enzyme *Bgl*II (Thermo Scientific) in fast digest buffer (FD buffer) provided by the manufacturer. Digestion was done at 37°C for 30 minutes in a total digest volume of 30 µL. After digestion the enzyme was inactivated at 80°C for 10 minutes. WalkOL-*Bgl*II cassette (“WalkOL” +”RGW-*Bgl*II“ oligos) was ligated to the digested sample in the following mixture: 15 µL *Bgl*II digested sample (fifth enrichment), 0.4 µL (0.5 pmol/µL) WalkOL-*Bgl*II, 3 µL (10x) T4 Ligase buffer, 1 µL T4 Ligase (5 U/µL) and 10.6 µL NF water (Sigma). The ligation process was set to run at 16°C overnight and was thereafter kept at 4°C. PCR was performed using (1 pmol/µL) WalkID primer and (10 pmol/µL) HPV5994r using DreamTaq polymerase (Thermo Scientific). PCR conditions were 35 cycles at temperatures of 94°C/64.4°C/72°C and at time intervals of 15sec/15sec/90sec. A specific PCR product was excised from a 1% agarose gel and was purified using GeneJet Gel Extraction Kit (Thermo Scientific). The eluted product was re-amplified using PCR conditions identical to those described above with the only change being that 10 pmol/µL WalkID primer was added instead of 1 pmol/µL. The sample was purified to remove excess primers and nucleotides and was sequenced by Eurofins MWG (Germany) using HPV5994r and WalkID primers. The RGW procedure was repeated using *Hind*III for digestion, a “WalkOL + *RGW-HindIII*“ cassette and primer HPV7459f instead of HPV5994r.

### PCR for sequence assembly

PCR was carried out by mixing the following: 2.5 µL (10x) reaction buffer, 2.5 µL 2 mM dNTP, 2.5 µL 25 mM magnesium, 1 µL forward primer, 1 µL reverse primer, 1 µL BSA, 0.2 µL DreamTaq polymerase (Thermo Fisher Scientific) and NF water to a total volume of 25 µL. Amplification was carried out using 35 cycles at temperatures of 94°C/60°C/72°C and time intervals of 15 sec/15 sec/90 sec. Two µL PCR product was loaded to 1% agarose gels.

### Sequence Assembly, Blast and alignment

Assembly of both RGW-sequences and PCR from forward and reverse sequencing was done using the assembly function of “CLC Main Workbench” with a minimum requirement of 30 bp overlap. The assembled sequence was blasted against the GenBank database [15], and most related sequences from the database were downloaded for alignment analysis.

## Results

### PINS Enrichment

In order to demonstrate enrichment of an extremely rare target using PINS, HPV18 containing DNA was mixed into a target-negative human background at a ratio of 1∶100,000 or 0.001% (w/w). 10 individual 1 µL samples of the mix were amplified using Phi29 amplification. The Phi29 amplified products were easily recognized as smears on a 0.7% agarose gel resembling those described using Exo-resistant primers in [16]. To identify amplified samples containing the HPV18 target, PCR was performed using primers HPV5901f and HPV5994r detecting a 113 bp fragment in the L1 gene of HPV18.

One sample out of ten was found to have an increased amount of HPV18 target, as analyzed by MPN5. Further analysis showed that the positive sample contained 30 HPV18 targets per µL corresponding to 0.1 targets/ng total DNA or approximately 50-fold more than the original sample. The results from all six rounds of PINS enrichment can be found in Table 2.

The procedure was repeated six times with the only modification being that the amplified sample, with progressively higher target copy number, was diluted more for each repetition. Thus, the second round of enrichment was initiated from the sample selected from the first round of enrichment by dilution until on average one to two Phi29 amplified products resulted in detection of the target HPV DNA after Phi29 amplification. 1 µL diluted template was added to each of the 10 Phi29 amplifications. The second round of amplification resulted in 3 positive samples from where the HPV18 DNA could be detected by PCR. Of these three, one sample was markedly higher in copy number than the two others. Consequently, only the sample with the highest quantity of targets was selected for further analysis. MPN5 from this sample was determined to be 170 targets/µL, corresponding to 0.8 targets/ng total DNA or approximately 280 fold higher than the original sample. Subsequently, the procedure was repeated four additional times resulting in a final target concentration of 33000 copies/µL or 786 targets/ng. In total, the HPV18 containing fragment was enriched more than 275,000 fold as compared to the original sample.

### Rapid genome walking (RGW) and sequence analysis of the enriched sample

At an abundance of 786 targets/ng, the final sample could have been sequenced directly using next generation sequencing technology. However, even the PINS round 5 sample could be sequenced using the rapid genome walking method [14]. In RGW, the DNA is fragmented using a restriction enzyme and linkers are ligated to the ends of the fragment. PCR products are then produced using an internal primer and a linker primer and the fragments are sequenced by Sanger sequencing.

**Figure 2:**
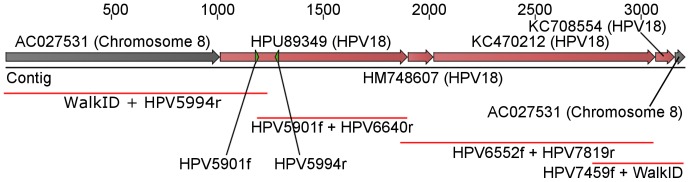
Alignment of RGW sequence and HPV specific sequencing from this study to published sequences on human chromosome 8 and HPV18 sequences. Positions of the primers used for detection of the HPV18 target sequence in the enrichment (HPV5901f and HPV5994r) are illustrated by small triangles in HPU89349.

Two PCR products spanning the 5′ and 3′ integration site of HPV18 respectively were produced from the sample selected in PINS enrichment round 5 and sequenced using RGW (“WalkID + HPV5994r” and “HPV7459f + WalkID”, Figure 2). Two additional PCR products “HPV5901f + HPV6640r” and “HPV6552f + HPV7819r” were produced from the same sample to cover the remaining HPV18 sequence. The PCR products were sequenced in both directions. The eight sequences were assembled into one continuous sequence of 3208 base pairs (Table S1).

Compared to the data available in GenBank, position 1–1011 of the assembled sequence aligned with 99.1% identity to chromosome 8 of the human genome sequence (GenBank accession number: AC027531). Position 3158–3208 aligned with 100% identity to the same region of chromosome 8, 3441 base pairs downstream of the 5′ integration point (GenBank accession number: AC027531). The less than 100% identity (99.1%) in the upstream sequence was due to an 8 bp deletion. Sequencing of a pure HeLa DNA sample as provided from NEB revealed the same deletion and the deletion was therefore not introduced during PINS enrichment.

The 2147 base pair fragment from position 1012 to 3158 aligned with 100% identity to published HPV18 sequences (Genbank accession numbers: HPU89349, HM748607, KC470212, and KC708554). A graphical representation of the assembly and associated GenBank sequences can be seen in Figure 2.

## Discussion

In this study we present a novel method for enrichment of DNA fragments based on minimal sequence information. The enrichment can be performed on very low abundance target DNA, in this case down to 0.0028 copies of target per ng of total DNA, and is generally applicable for enrichment of DNA fragments from complex samples. It is shown that an HPV18 fragment can be enriched more than 275,000 fold through six rounds of enrichment thereby enabling sequencing of a fragment of more than 3000 bp fragment containing a short known sequence used to direct the enrichment.

The enrichment in each round of PINS ranged from a 4.2-fold to a 45-fold increase (targets per ng total DNA) with an average of 12 fold, and the degree of enrichment in each round correlated to the ratio of positive to negative wells as expected. After six rounds of amplification, the sample contained 2.8 targets per human genome (abundance of 275%) and sequencing was thereby enabled. Rapid genome walking and PCR employing approximately 1000 bp fragments could be performed from samples with at least 0.44 targets per genome, i.e. from PINS rounds 5 and 6. At target concentrations below this level, no larger PCR fragments were obtained and sequencing could therefore not be performed (data not shown).

Using the relative simple RGW method it was shown that, although the DNA region of interest had been amplified through five consecutive rounds of PINS, the sequences from the pure HeLa sample and the enriched fragment were 100% identical and the HPV18 sequence was identical to previously published sequences. This observation correlates well with the low error rate previously reported for Phi29 [17], and is of high importance for the final evaluation of the sequences.

Although the enrichment was based on PCR detection of a short specific PCR fragment of only 113 base pairs it resulted in enrichment of a much larger genome fragment as determined by sequencing. The data analysis of the present study describes the exact integration site of HPV18 into human chromosome 8 including 1026 base pairs of upstream sequence and correlating to identical findings in previous studies [18]. To further analyze the enriched sequence, an additional DNA sequence of 2182 base pairs was amplified, sequenced and analyzed confirming the integration site in chromosome 8 and showing 2147 bp of the integrated HPV18 virus. Phi29 is known to produce fragments of an average length of approximately 70 kb [19] and it is therefore likely that the enriched fragment is significantly longer than the 3208 bp reported here. The exact length of the fragment could possibly be deduced from next generation sequencing.

Earlier publications have pointed out, that amplification bias is an inherent feature of Phi29 amplification, and most reactions mixtures are known to produce DNA solely from the kit components [20]. The phenomenon is generally described as non-template-reactions (NTR) or template-independent products (TIPs), and various attempts have been introduced to circumvent the problem [21]. Phi29 kits producing no NTR are commercially available, as exemplified by the Replig-g Ultra-Fast mini kit (Qiagen), but the requirement for template quantity in such kits is typically high (>10 ng), leaving amplification of very dilute samples impossible. In the current study we have shown that the applied reaction mixtures provided an efficient amplification with templates as dilute as 8 pg corresponding to less than 2.5 human genomes, without observing any reaction in the negative control (NTR) and without requirement of additional UV-treatment [20] or addition of inhibitory compounds [21]. Moreover, no amplification errors were observed after six rounds of amplification.

Regardless of application, PINS provides a powerful method to gain sequence information from a minute sub-fraction of a complex sample with a minimum of prior sequence information. In the present example, at least 3208 base pair of sequence was enriched from a very complex background based on just 39 base pairs of sequence information (the two detection primers).

The current example is based on an integrated virus in a complex background and may be used to determine other virus sequences and integration sites. However, we also see an obvious possibility to broaden the usage to include applications comprising other partially unknown sequences in complex samples such as cells or DNA in blood samples, swabs, biopsies, feces samples or complex environmental samples, thereby enabling sequencing or dramatically reducing the volume of sequencing reads.

## Supporting Information

Table S1 **Assembled sequence of the enriched HPV18 fragment.**
(DOCX)

## References

[1] Bidard FC, Weigelt B, Reis-Filho JS (2013) Going with the flow: from circulating tumor cells to DNA. Sci Transl Med 5: 207ps214. 10.1126/scitranslmed.3006305 24132635

[2] Gibbons JG, Janson EM, Hittinger CT, Johnston M, Abbot P, et al (2009) Benchmarking next-generation transcriptome sequencing for functional and evolutionary genomics. Mol Biol Evol 26: 2731–2744. 19706727 10.1093/molbev/msp188

[3] Milbury CA, Li J, Makrigiorgos GM (2011) Ice-COLD-PCR enables rapid amplification and robust enrichment for low-abundance unknown DNA mutations. Nucleic Acids Res 39: e2. 20937629 10.1093/nar/gkq899 PMC3017621

[4] Koboldt DC, Steinberg KM, Larson DE, Wilson RK, Mardis ER (2013) The next-generation sequencing revolution and its impact on genomics. Cell 155: 27–38. 24074859 10.1016/j.cell.2013.09.006 PMC3969849

[5] Sykes PJ, Neoh SH, Brisco MJ, Hughes E, Condon J, et al (1992) Quantitation of targets for PCR by use of limiting dilution. Biotechniques 13: 444–449. 1389177

[6] Dean FB, Hosono S, Fang L, Wu X, Faruqi AF, et al (2002) Comprehensive human genome amplification using multiple displacement amplification. PNAS 99: 5261–5266. 11959976 10.1073/pnas.082089499 PMC122757

[7] Myers LE, McQuay LJ, Hollinger FB (1994) Dilution assay statistics. J Clin Microbiol 32: 732–739. 8195386 10.1128/jcm.32.3.732-739.1994 PMC263116

[8] Vogelstein B, Kinzler KW (1999) Digital PCR. PNAS 96: 9236–9241. 10430926 10.1073/pnas.96.16.9236 PMC17763

[9] Bosch FX, Lorincz A, Munoz N, Meijer CJ, Shah KV (2002) The causal relation between human papillomavirus and cervical cancer. J Clin Pathol 55: 244–265. 11919208 10.1136/jcp.55.4.244 PMC1769629

[10] Gillison ML, Koch WM, Capone RB, Spafford M, Westra WH, et al (2000) Evidence for a causal association between human papillomavirus and a subset of head and neck cancers. J Natl Cancer Inst 92: 709–720. 10793107 10.1093/jnci/92.9.709

[11] Li N, Franceschi S, Howell-Jones R, Snijders PJ, Clifford GM (2011) Human papillomavirus type distribution in 30,848 invasive cervical cancers worldwide: Variation by geographical region, histological type and year of publication. Int J Cancer 128: 927–935. 20473886 10.1002/ijc.25396

[12] Schneider UV, Mikkelsen ND, Lindqvist A, Okkels LM, Johnk N, et al (2012) Improved efficiency and robustness in qPCR and multiplex end-point PCR by twisted intercalating nucleic acid modified primers. PLoS One 7: e38451. 22701644 10.1371/journal.pone.0038451 PMC3368873

[13] Oblinger J, Koburger J (1975) Understanding and teaching the most probable number technique. J Milk Food Technol 38: 540–545.

[14] Kilstrup M, Kristiansen KN (2000) Rapid genome walking: a simplified oligo-cassette mediated polymerase chain reaction using a single genome-specific primer. Nucleic Acids Res 28: e55. 10871354 10.1093/nar/28.11.e55 PMC102640

[15] Benson DA, Karsch-Mizrachi I, Lipman DJ, Ostell J, Wheeler DL (2005) GenBank. Nucleic Acids Res 33 Database Issue: D34–D38. 10.1093/nar/gki063 PMC540017 15608212

[16] Dean FB, Nelson JR, Giesler TL, Lasken RS (2001) Rapid amplification of plasmid and phage DNA using Phi 29 DNA polymerase and multiply-primed rolling circle amplification. Genome Res 11: 1095–1099. 11381035 10.1101/gr.180501 PMC311129

[17] Han T, Chang CW, Kwekel JC, Chen Y, Ge Y, et al (2012) Characterization of whole genome amplified (WGA) DNA for use in genotyping assay development. BMC Genomics 13: 217. 22655855 10.1186/1471-2164-13-217 PMC3403925

[18] Landry JJ, Pyl PT, Rausch T, Zichner T, Tekkedil MM, et al (2013) The genomic and transcriptomic landscape of a HeLa cell line. G3 (Bethesda) 3: 1213–1224. 23550136 10.1534/g3.113.005777 PMC3737162

[19] Lasken RS, Huges S (2005) Multiple displacement amplification of genomic DNA. In: Huges S, editor. Whole Genome Amplification. Oxfordshire: Scion Publishing Ltd. 2005. pp.99–118.

[20] Woyke T, Sczyrba A, Lee J, Rinke C, Tighe D, et al (2011) Decontamination of MDA reagents for single cell whole genome amplification. PLoS ONE 6: e26161. 22028825 10.1371/journal.pone.0026161 PMC3197606

[21] Pan X, Urban AE, Palejev D, Schulz V, Grubert F, et al (2008) A procedure for highly specific, sensitive, and unbiased whole-genome amplification. PNAS 105: 15499–15504. 18832167 10.1073/pnas.0808028105 PMC2563063

