# Supplementary material for: Partition Enrichment of Nucleotide Sequences (PINS) - A Generally Applicable, Sequence Based Method for Enrichment of Complex DNA Samples
Source: PLoS One. 2014 Sep 9;9(9):e106817. doi: 10.1371/journal.pone.0106817 (PMC4159240; doi:10.1371/journal.pone.0106817)
Supplement: Table S1 — Assembled sequence of the enriched HPV18 fragment. (DOCX) [file pone.0106817.s001.docx]

**Supporting information Table S1**

| Sequence ID | Nucleotide sequence (5’ – 3’) |
| --- | --- |
| >Contig (3208bp) | AACACTACTGATTCAGACATTAATCTTAAGTATCCAGGGAGCCCTAAAACATTGTATCCCACTAGCAAGGACCATGGTAATTGCCACGTAAATCCCCTCCATTATGTGGCCCTTATTATGACCAGCCAGCCAAGGCTTGCCTTTAAATCATACCAATTGAACCGAGCCTTGTAGAAACACTATCACCTACGCATACCTCTGCTTCTTTTCATTAACCTGCTATCCTCTTTACAAATGGGATTCTTCACCCACTCCCTTCTTCTAGATTAGCAATGCCCTGTTAAGTAAACGAACACGAAATTCAAAGGGAAACAGGAGCAATCATCATTACCAGCTGCCGTGTTAAGCATTGCGAAAACGCTCACGATTCACAGAAAAATCCATGCTGTTCTTTGAAGGCATTCAAGCCTTAATAGCTAGCTGGATGAATGTTTAACTTCTAGGCCAGGCACTACTCTGTCCCAACAATAAGCCCTGTACATTGGGAAAGGTGCCGAGACATGAACTTTGGTCTTCTCTGCAATCCATCTGGAGCATTCACTGACAACATCGACTTTGAAGTTGCACTGACCTGGCCAGCCCTGCCACTTACCAGGTTGGGTAAGTTATTCATTTCTTTATTAATTCATTCATTCATTCAATTTATGGGTATTTTGTGTCATACACTTGCTAGGCACAACATATATATATATGTGTGTGTATATATATGTGTGTGTGTGTGTGTATATATATACACACACACATATATAAAATCAGTAAAGTAATGAGAACTCAGAAAAAGAGCAATTTAACCAGAATTGGAAGGTCAAGGAGGGCCGCCTGGAGGAAACAATATCTAAGCTAAAACATGGAGACTGAGGAGTATGAGGCCAAATTTTCTCTTCTGTGAAATGGGCATGATGCTCTCTTGAAAAGCAGTAGTGATGTTAAGTCAGATGATTTTCTTAAAATGGACAATGGCTTTGGACTCCGATTTGGCATTTGACAAATATTATTAAAGTTTTTGTCTGGTTATTAACTGTTGGTAATCCATATTTTAGGGTTCCTGCAGGTGGTGGCAATAAGCAGGATATTCCTAAGGTTTCTGCATACCAATATAGAGTATTTAGGGTGCAGTTACCTGACCCAAATAAATTTGGTTTACCTGATAATAGTATTTATAATCCTGAAACACAACGTTTAGTGTGGGCCTGTGCTGGAGTGGAAATTGGCCGTGGTCAGCCTTTAGGTGTTGGCCTTAGTGGGCATCCATTTTATAATAAATTAGATGACACTGAAAGTTCCCATGCCGCCACGTCTAATGTTTCTGAGGACGTTAGGGACAATGTGTCTGTAGATTATAAGCAGACACAGTTATGTATTTTGGGCTGTGCCCCTGCTATTGGGGAACACTGGGCTAAAGGCACTGCTTGTAAATCGCGTCCTTTATCACAGGGCGATTGCCCCCCTTTAGAACTTAAAAACACAGTTTTGGAAGATGGTGATATGGTAGATACTGGATATGGTGCCATGGACTTTAGTACATTGCAAGATACTAAATGTGAGGTACCATTGGATATTTGTCAGTCTATTTGTAAATATCCTGATTATTTACAAATGTCTGCAGATCCTTATGGGGATTCCATGTTTTTTTGCTTACGGCGTGAGCAGCTTTTTGCTAGGCATTTTTGGAATAGGGCAGGTACTATGGGTGACACTGTGCCTCAATCCTTATATATTAAAGGCACAGGTATGCGTGCTTCACCTGGCAGCTGTGTGTATTCTCCCTCTCCAAGTGGCTCTATTGTTACCTCTGACTCCCAGTTGTTTAATAAACCATATTGGTTACATAAGGCACAGGGTCATAACAATGGTGTTTGCTGGCATAATCAATTATTTGTTACTGTGGTAGATACCACTCGCAGTACCAATTTAACAATATGTGCTTCTACACAGTCTCCTGTACCTGGGCAATATGATGCTACCAAATTTAAGCAGTATAGCAGACATGTTGAGGAATATGATTTACAGTTTATTTTTCAGTTGTGTACTATTACTTTAACTGCAGATGTTATGTCCTATATTCATAGTATGAATAGCAGTATTTTAGAGGATTGGAACTTTGGTGTTCCCCCCCCGCCAACTACTAGTTTGGTGGATACATATCGTTTTGTACAATCTGTTGCTATTACCTGTCAAAAGGATGCTGCACCGGCTGAAAATAAGGATCCCTATGATAAGTTAAAGTTTTGGAATGTGGATTTAAAGGAAAAGTTTTCTTTAGACTTAGATCAATATCCCCTTGGACGTAAATTTTTGGTTCAGGCTGGATTGCGTCGCAAGCCCACCATAGGCCCTCGCAAACGTTCTGCTCCATCTGCCACTACGTCTTCTAAACCTGCCAAGCGTGTGCGTGTACGTGCCAGGAAGTAATATGTGTGTGTGTATATATATATACATCTATTGTTGTGTTTGTATGTCCTGTGTTTGTGTTTGTTGTATGATTGCATTGTATGGTATGTATGGTTGTTGTTGTATGTTGTATGTTACTATAATTGTTGGTATGTGGCATTAAATAAAATATGTTTTGTGGTTCTGTGTGTTATGTGGTTGCGCCCTAGTGAGTAACAACTGTATTTGTGTTTGTGGTATGGGTGTTGCTTGTTGGGCTATATATTGTCCTGTATTTCAAGTTATAAAACTGCACACCTTACAGCATCCATTTTATCCTACAATCCTCCATTTTGCTGTGCAACCGATTTCGGTTGCCTTTGGCTTATGTTTGTGGTTTTCTGCACAATACAGTACGCTGGCACTATTGCAAAATTTAATCTTTTGGGCACTGCTCCTACATATTTTGAACCATTGGCGCGCCTCTTTGGCGCATACAAGGCGCACCTGGTATTAGTCATTTTCCTGTCCAGGTGCGCTACAACAATTGCTTGCATAACTATATCCACTCCCTATGTAATAAAACTGCTTTTAGGCACATATTTTAGTTTGTTTTTACTTAAGCTAATTGCATACTTGGCTTGTACAACTACTTTCATGTCCAACATTCTGTCTACCCTTAACATGAACTATAATATGACTAAGCTGTGCATACATAGTTTATGCAACCGAAATAGGTTGGGCAGCACATACTATACTTTTCATTAATACTTTTAACAATTGTAGTAAACTTAGCTTCTTGCTCGTCTCATGGTTGCTTTTTTTTCTTTTTCCTTTT |
